# Relations of problematic online dating app use with mental and sexual health: a cross-sectional study in Swiss university students

**DOI:** 10.1136/bmjph-2025-002569

**Published:** 2025-09-08

**Authors:** Babette L. Winter, Clarissa Janousch, Fabienne S. V. Wehrli, Jan S. Fehr, Benjamin Hampel, Boris B. Quednow

**Affiliations:** 1 Department Public and Global Health, Institute of Epidemiology Biostatistics and Prevention, University of Zurich, Zurich, Switzerland; 2 Experimental Pharmacopsychology and Psychological Addiction Research, Department of Adult Psychiatry and Psychotherapy, Psychiatric University Hospital Zurich, Zurich, Switzerland; 3 University of Zurich Jacobs Center for Productive Youth Development, Zurich, Switzerland; 4 Clinical Psychology for Children/Adolescents and Couples/Families, Department of Psychology, University of Zurich, Zurich, Switzerland; 5 Checkpoint Zurich, Zurich, Switzerland; 6 Neuroscience Center Zurich, Zürich, Switzerland

**Keywords:** Cross-Sectional Studies, Depression, Mental Health, Public Health, Sexual Health

## Abstract

**Background:**

The rise of online dating apps (ODAs) has raised concerns about potential mental and sexual health risks, particularly among emerging adults. However, limited research exists on how problematic ODA use and health outcomes are associated during this sensitive transition phase of early adulthood.

**Methods:**

Data were collected in an anonymous online survey from 923 Swiss university students (64% female) who had actively used ODA in the past 12 months. The Problematic Online Dating Apps Use Scale (PODAUS) and a variety of mental health, substance use and sexual health outcomes were assessed. Regression models were applied to examine associations between problematic ODA use and health outcomes. Measurement invariance (MI) of the PODAUS was tested across females and males. In a subsample (*N*=275), the PODAUS was retested at a 14-day follow-up.

**Results:**

Higher intensity of problematic ODA use was significantly associated with more symptoms of depression, impulsivity and a higher number of sexual partners. Furthermore, a higher PODAUS score was associated with higher lifetime and 12-month prevalence rates of sexually transmitted infections. PODAUS demonstrated good internal consistency (α=0.73) and acceptable retest reliability (r_s_=0.54**), while MI was confirmed across genders.

**Conclusions:**

We identified significant associations between problematic ODA use and adverse health outcomes in a student population. Our findings highlight the necessity for targeted mental health interventions and sexual health education for university students, especially for those students exhibiting problematic ODA use patterns.

**WHAT IS ALREADY KNOWN ON THIS TOPIC**

**WHAT THIS STUDY ADDS**

- This study demonstrates that problematic ODA use is associated with increased symptoms of depression, impulsivity and risky sexual behaviours, such as a higher number of sexual partners and elevated prevalence of sexually transmitted diseases, in university students.

**HOW THIS STUDY MIGHT AFFECT RESEARCH, PRACTICE OR POLICY**

- Our findings highlight the need for targeted mental and sexual health interventions for university students who engage in problematic ODA use. Future research should further explore the long-term effects and causal pathways of these associations.

## Introduction

 The rise of online dating apps (ODAs) has fundamentally changed how individuals meet and form romantic and sexual relationships. With over 300 million global ODA users in 2023 and expected upward trends, according to Business of Apps’ Dating app report, meeting online has become the new norm for people to connect.^1 2^ While these platforms offer unprecedented opportunities for social and sexual connection, concerns have arisen regarding their potential addictive effects and their putative impact on mental health, substance use and sexual health.^3^ Understanding these associations is critical, particularly for vulnerable populations such as young adults, who tend to engage in risky behaviours during the developmental phase of emerging adulthood (ages 18–24).^4 5^

### Emerging adulthood and risk behaviours

During emerging adulthood, individuals face essential developmental transitions, including identity formation, autonomy and establishing intimate relationships.^4 5^ This period is pivotal as students are expected to manage shifts in education, employment and interpersonal relationships, laying the base for lifelong well-being while facing heightened susceptibility to health adversities.^6 7^

At the same time, the brain is undergoing accelerated development and is at greater sensitivity to risk exposures commonly encountered by young adults, including illegal substance use, alcohol bingeing, sleep disruption and possibly also digital influences.^8^ Silvers *et al., 2023*^7^ noted that reward-motivated behaviours during emerging adulthood are crucial as individuals must take risks and explore to become increasingly self-sufficient. In this review, they concluded that high reward sensitivity can lead to risky decision-making and substance use disorders, while low reward sensitivity can result in depression. Given that reward sensitivity peaks during emerging adulthood, ODAs might have high addictive potential in this vulnerable demographic, given their inherent rewarding properties.

### Mental and sexual health implications

The mental health and well-being of university students have emerged as significant public health concerns, as most mental disorders tend to manifest during early adulthood.^9 10^ In a survey by the WHO, it was found that substance use, depression and anxiety disorders are particularly prevalent among emerging adults, with research indicating a 12-month prevalence of 20% of diagnosed psychiatric disorders in students aged 18–22.^11^ Furthermore, a recent publication reported a depression prevalence of 25% among university students, which is double the rate observed in the general population.^12^

Young adults also explore their sexuality, often engaging in higher-risk sexual behaviours such as having multiple partners, which can increase the likelihood of contracting sexually transmitted infections (STIs).^13^,^15^ Thus, students have to confront myriad challenges, including peer pressures as well as mental and sexual health issues, with the potential for exacerbated consequences due to ODA use.^16^

### Advantages and risks of ODA use

In contrast to conventional web-based online dating sites, smartphone-based ODAs have been tailored to young adults, with features like geosocial networking enhancing accessibility and prolonged engagement.^17 18^ One of the advantages of ODAs is their ability to facilitate social connections among university students during this critical period of personal and social development. These platforms provide an accessible means of meeting new people that transcends traditional barriers of time and location, allowing users to interact with potential partners conveniently, which can be particularly advantageous for those who may experience social anxiety or have limited access to traditional dating venues.^19^ These apps have become increasingly accessible, acceptable and affordable, making the younger generation particularly susceptible to excessive and problematic use.^17^ Problematic ODA use is conceptualised based on Griffiths’ Components Model of Addiction and is adapted from the definition of problematic social networking sites use.^20 21^ Thus, problematic ODA use is defined as an intense preoccupation with ODAs, characterised by excessive time spent on them, that conflicts with other essential life domains, such as social activities, interpersonal relationships and psychological well-being, along with repeated unsuccessful attempts to reduce usage. Indeed, young people’s online time has doubled in the past decade.^22^ A recent study indicated that 18–29 year-olds are the primary age group of ODA users.^23^

Thus, during this phase of developmental transitions, adverse effects of problematic ODA use on students’ health and well-being might be especially pronounced. There is a scarcity of studies investigating problematic ODA use and health outcomes in emerging adults. When investigating ODA use in general, existing studies indicate that ODA use is associated with poorer mental health in young adults, including heightened social anxiety, symptoms of depression,^24 25^ increased psychosocial stress in students^26^ and elevated depression in recent but not lifetime ODA use.^27^

In a large-scale longitudinal study assessing Norwegian university students, Tinder use was associated with increased anxiety symptoms, hazardous alcohol use, substance use and risky sexual behaviour.^28^ Additionally, several studies found that young adult ODA users present higher scores of sexual addiction, higher unsafe sexual practices and more risky sexual behaviour, including a higher number of sexual partners.^28^,^32^ This raises the question of whether emerging adults using ODAs are at a higher risk for contracting STIs. This existing literature on the impact of ODA use on health outcomes has predominantly focused on comparisons between users and non-users, often neglecting the intensity and problematic nature of ODA use. Furthermore, the relationship between problematic ODA use and outcomes related to mental and sexual health has not been extensively studied in university students. Finally, previous investigations into problematic ODA use have typically focused on single-app contexts, such as the Problematic Tinder Usage Scale (PTUS).^31 33^ Research investigating problematic ODA use across multiple ODAs has only recently become apparent using the recently developed Problematic Online Dating App Usage Scale (PODAUS).^34 35^ Using this scale, recent findings have linked problematic ODA use to adverse mental health outcomes in middle-aged, HIV-negative men-having-sex-with-men (MSM), including increased symptoms of depression, anxiety, impulsivity and attention deficit hyperactivity disorder.^35^ However, whether these findings can be confirmed in emerging adults remains unclear.

### Study objectives

In the present study, we aim to investigate the relationship between problematic ODA use and mental health, substance use and sexual health and behaviour in a large sample of Swiss university students. Based on recent findings on these theoretical frameworks, we expect that higher intensity of problematic ODA use is associated with elevated symptoms of depression, higher impulsivity, higher substance use rates, higher risky sexual behaviour and adverse sexual health effects. Additionally, the test-retest reliability and measurement invariance (MI) of the PODAUS across genders will be assessed.

## Materials and methods

We followed the ‘Strengthening the Reporting of Observational Studies in Epidemiology’ (STROBE) statement for reporting observational studies.^36^

### Study design and setting

Data for this anonymous online survey were assessed cross-sectionally (PODAUS and health outcomes) and longitudinally (PODAUS test–retest reliability at 2 weeks) and obtained through an anonymous online survey using the electronic data capture tool software REDCap.^37^ The survey was conducted between January and May 2023 at the University of Zurich, Switzerland. Participants were recruited by convenience sampling through the university’s email in two distinct recruitment waves and through the snowballing principle to maximise outreach and ensure a representative student population aggregated into a single sample.

### Study participants

Participants were eligible for participation if they (a) were aged ≥18 years, (b) were students at the University of Zurich, (c) used ODAs regularly in the past 12 months and (d) were German-speaking. ODAs were defined as all types of GPS-based social networking apps that run on smartphones and are used to find potential relationships or sexual partners. Potential participants were excluded if they did not complete the PODAUS or were older than 39 years.

### Procedure and measures

In addition to the PODAUS, participants answered questionnaires on mental health, sexual behaviour and substance use at baseline (see below). Survey completion took an average of 30 min. All included questionnaires were self-reporting instruments, with higher scores indicating higher levels of psychopathology (see online supplemental table SI for internal consistency of all scales). Back-translation processes were employed for scales unavailable in German.^38^ The follow-up questionnaire was sent exclusively to participants of the second data collection wave.

#### Problematic ODA use

Participants were asked to specify which ODAs they used, with the option to select multiple apps. The listed apps were identified through a literature review, and participants could also include additional ODAs not listed by providing a free-text entry. Problematic ODA use was assessed with the 6-item German PODAUS^35^ (original Italian version by Gori *et al*).^34^ Participants responded on a 4-point scale ranging from 1 (do not agree at all) to 4 (agree completely; see online supplemental table SII for a German and online supplemental table SIII for English PODAUS version). Weekly time spent on ODAs (hour/week) was assessed by summing reported weekday and weekend day usage time. Participants were also asked additional exploratory binary questions (y/n) regarding their ODA use. These asked if participants had (1) ever experienced stalking on ODAs, (2) been offered illegal substances for sale, (3) started taking illegal substances because of their ODA use, (4) taken more illegal substances because of their ODA use, (5) ever deleted and reinstalled ODAs and (6) if so, how often. Problematic Tinder use was assessed with a translated German version of the original 6-item *PTUS* on a 5-point Likert scale.^33^

#### Mental health

Depressive symptoms in the past 14 days were assessed using the 9-item Patient Health Questionnaire, scored on a 4-point Likert scale.^39^ Trait Impulsivity was assessed using the 15-item Barratt Impulsiveness Scale (BIS-15) on a 4-point Likert scale.^40^

#### Substance use

The intensity of alcohol intake was assessed using the 10-item Alcohol Use Disorder Identification Test self-reporting screening on a 5-point Likert scale.^41^ For nicotine use, the 12-month prevalence, as well as daily frequency (number of cigarettes per day averaged over the 30 days), was assessed. Lifetime illegal substance use was measured with a single item (y/n/do not want to say). For the substances cannabis, cocaine, Ecstasy (3,4-methylenedioxymethamphetamine, MDMA) and (meth-)amphetamine, participants reported the 12-month prevalence (y/n/do not want to say) and 30-day frequency of consumption events. We focused on the most frequently used substances according to previous data from a Zurich young adult cohort with addictive potential.^42 43^

#### Sexual behaviour and sexual health

Participants were asked if they had sexual intercourse in the past 3 months (y/no/do not want to say). The number of sexual partners was assessed using an ordinal scale: 0–5 partners corresponded to the exact number, and 6 indicated more than five partners. Sexual self-esteem was assessed using the 8-item Sexual Self-esteem Scale on a 5-point Likert scale.^44^ Sexual health was assessed by asking participants’ lifetime and 12-month prevalence of STIs, along with the specific diagnoses they received. Sex life happiness was assessed using a continuous item ranging from 0 (not at all) to 100 (very).

### Statistical analysis

To assess associations between PODAUS scores and risk outcomes for mental health, substance use and sexual behaviour, Spearman’s rank correlations (r_s_) were performed. Multiple regression analyses were conducted to examine PODAUS’s predictive role on mental health, sexual health and substance use outcomes with the covariates age and gender for each model. Due to the different data types of models, unstandardised estimates (B) are reported. Ordinary least squares regression was used for continuous outcomes, logarithmic regression for binary outcomes and ordinal outcomes, and weighted least squares regression for binary outcomes, addressing heteroscedasticity and non-normality.^45^ Effect sizes for continuous outcomes were interpreted using Cohen’s f² (f²=0.02 small, f²=0.15 medium, f²=0.35 large), and ORs were calculated for binary outcomes and incidence rate ratios for count data.^46 47^ A significance threshold of p<0.01 was set in multiple linear regression analysis to avoid **Type** I error inflation and correct for multiple testing. MI of the PODAUS was assessed across genders at three levels (configural, metric and scalar) using multigroup confirmatory factor analyses (CFAs), following official recommendations.^48 49^ Invariance was evaluated using acceptable goodness-of-fit indices for configural models and changes in fit indices, with thresholds of ∆CFI≤0.01, paired with ∆SRMR≤0.030 for metric invariance and ∆SRMR≤0.015 for scalar invariance, as recommended by Chen.^50^ Reliability of the PODAUS was measured using Cronbach’s α and test–retest reliability at a 2-week interval.^51 52^ CFA and MI analyses were conducted using Mplus V.8.4,^53^ while all other analyses were performed with R Version 2023.06.1+524.^54 55^

### Patient and public involvement

Participants from the target population were consulted during the study design phase to identify relevant topics and ensure the study addressed their concerns. A prepiloting phase was conducted with participants from the target group to gather feedback on the survey content, clarity and estimated completion time. The public will be involved in the dissemination of findings through presentations and talks aimed at raising awareness and fostering discussion on the implications of problematic ODA use.

## Results

### Study sample

In total, 1906 individuals clicked on the survey link, 1905 took the survey and 923 participants fulfilled the inclusion criteria (see table 1 for descriptive statistics). Participants from the second recruitment wave (N=413) were contacted after 14 days for a retest of the PODAUS, with 275 being eligible. Most attrition was due to non-response rather than changes in eligibility, and the observed attrition rate of 17% is within the expected range for follow-up studies.^56^

**Table 1:** Descriptive statistics of demographics, ODA use, mental health, substance use and sexual health

		%	Md	IQR	Possible range
Demographics (n=923)					
Age			23.0	21–25	18–39
Sex assigned at birth	Female	66.5			
	Male	34.1			
	Other	0.4			
Gender	Female	64.1			
	Male	34.2			
	Non-binary	1.1			
	Other	0.6			
Sexual orientation	Heterosexual	72.2			
	Bisexual	16.9			
	Homosexual	5.7			
	Other	4.0			
	No information	1.2			
Relationship status	No partner	68.3			
	Monogamous	20.6			
	Open relationship	5.5			
	Other	4.1			
	Polyamorous	1.5			
Defined as minority	Yes	13.3			
	No	86.7			
Birth country	Switzerland	74.6			
	Other	16.0			
	Germany	9.4			
Residency	City	48.2			
	Agglomerations	30.1			
	Rural	21.7			
ODA use characteristics (N=923)					
Nr of ODAs used simultaneously			2.0	1–2	1–5
ODA preference	Tinder	77.4			
	Bumble	54.9			
	Hinge	14.3			
	Others	9.4			
	Grindr	5.7			
	OKCupid	5.2			
	PlanetRomeo	1.8			
ODA usage patterns	Used ODA last month	67.2			
	Used ODA last week	67.3			
	Used ODA today/yesterday	68.6			
Time spent on ODAs (in h)	Total weekly		2.33	1.2–4	0–37
	Weekday		0.33	0.2–0.5	0–5
	Weekend day		0.33	0.2–0.8	0–6
Motivations for use of favourite ODA	Finding a sex partner		4.0	2–4	1–5
	Finding a partner for life		4.0	3–4	1–5
	Confirmation for looks		3.0	1–4	1–5
	Chatting		3.0	2–4	1–5
	Escaping my problems		2.0	1–1	1–5
	Getting sex fast		2.0	1–3	1–5
	Afraid of missing a potential partner		2.0	1–3	1.5
	Masturbating		1.0	1–1	1–5
Problematic dating app use					
Baseline: Problematic Dating App Use (PODAUS; N=923)			9.0	7–11	6–24
Follow-up: Problematic Dating App Use (PODAUS; N=275)			8.0	6.5–10	6–24
Problematic Tinder Use (PTUS; n=617)			11.0	9–13	6–30
Additional ODA questions (%yes; N=923)	Experienced stalking on ODAs	9.9			
	Deleted and reinstalled ODAs	74.4			
	Illegal substances were offered for sale on ODAs	6.7			
	Started illegal substance use because of ODAs	1.5			
	More illegal substance use because of ODAs	3.9			
Mental health					
Depression (PHQ-9, n=911)	Total score	71.4			
	Mild signs of depression (score≥5)		7.0	4-11	0-27
Impulsivity (BIS-15, n=903)			28.0	24–33	14–70
Substance use					
Intensity of alcohol use (AUDIT, n=641)	Total score		2.0	1–4	0–40
	No signs (score<8)	91.4			
Suspicious signs (score≥8)	8.6				
Nicotine (12-month prevalence)	y,n	37.4			
Nicotine (cigarettes/day, n=890)			0	0–0	0–20
12-month prevalence (n=890)	y,n				
	Cannabis	35.6			
	Cocaine	7.5			
	Ecstasy/MDMA	8.0			
	(Meth-) Amphetamines	3.1			
30-day frequency (n=890)					
	Cannabis	0	0–0		0–30
	Cocaine	0	0–0		0–8
	Ecstasy/MDMA	0	0–0		0–4
	(Meth-) Amphetamines	0	0–0		0–30
Sexual health (N=923)					
Lifetime STI prevalence	y,n		0	0–0	0–3
	No STI	89.2			
	1 STI	10.0			
	2 STI	0.7			
	3 STI	0.1			
12-month STI prevalence	y,n		0	0–0	0–2
	No STI	96.1			
	1 STI	0.3			
	2 STI	0.2			
Sexual partner count (in past 3 months; n=867)			1.0	0–1	0–8
	0	38.6			
	1	39.9			
	2	10.1			
	3	6.8			
	4	1.5			
	5	0.8			
	>5	2.0			
	Did not want to say	0.3			
Sexlife happiness (n=923)			53.0	33–81	0–100
Sexual Self-esteem (SSES; n=885)			19.0	16–21	0–28

Due to the nature of online surveys and the non-forced response format, the number of participants for each instrument/question varied (please see the n as indicated in the table).

AUDIT, Alcohol Use Disorders Identification Test; BIS-15, Barratt Impulsiveness Scale; Md, median; MDMA, 3,4-methylenedioxymethamphetamine; ODA, online dating app; PHQ-9, Patient Health Questionnaire 9; PODAUS, Problematic Online Dating App Use Scale; PTUS, Problematic Tinder Use Scale; SSES, Sexual Self-Esteem Scale; STI, sexually transmitted infection.

**Figure 1:**
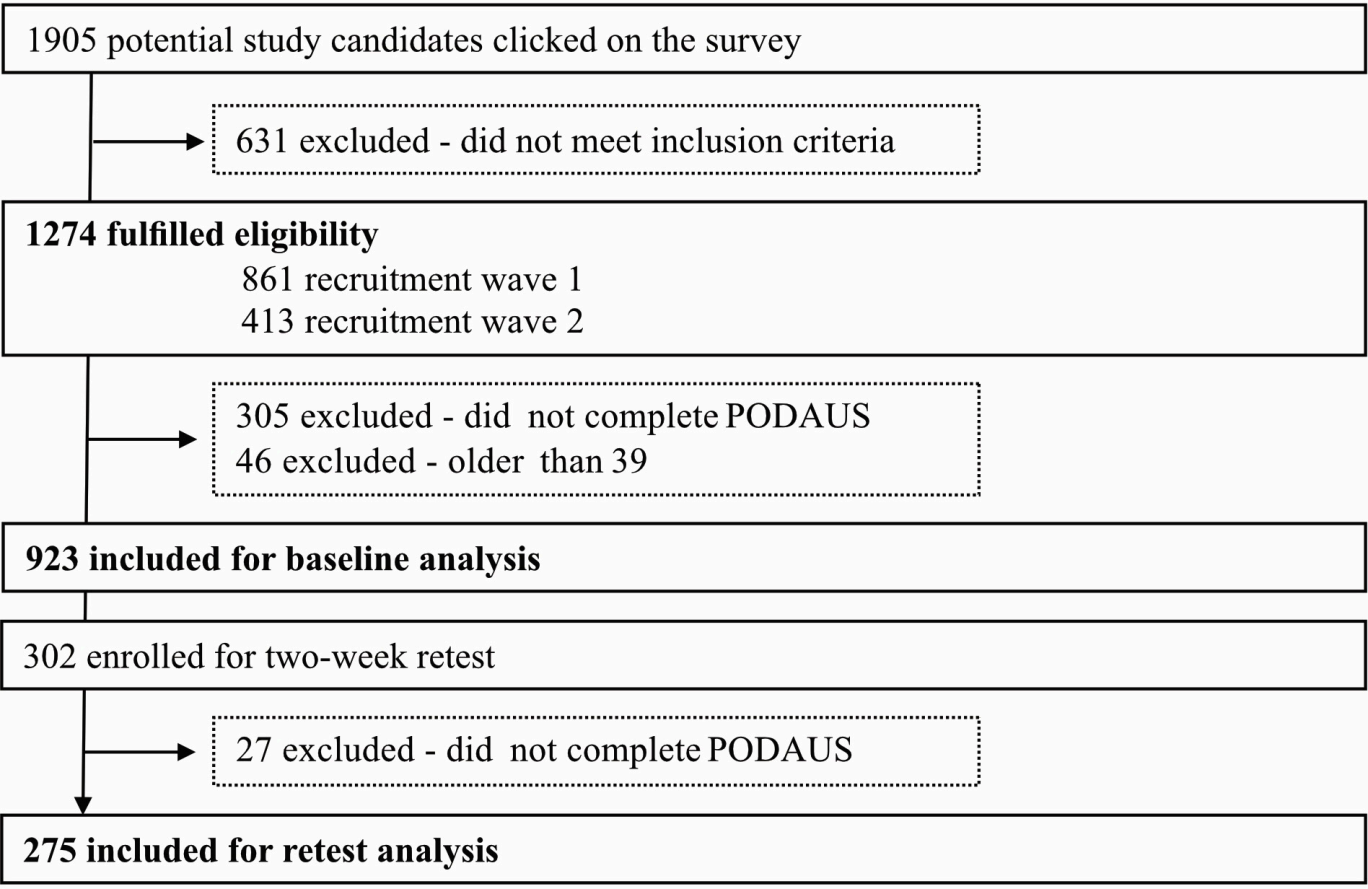
Study flowchart depicting participant inclusion and exclusion process. PODAUS, Problematic Online Dating App Usage Scale.

We conducted post hoc sensitivity analyses, which showed no significant differences between fast responders (<2 s per PODAUS item) and other participants; therefore, all participants were included in the analysis. Figure 1 depicts the inclusion process.

### Descriptive statistics

Participants were between 18 and 39 years old, with a median age of M_d_=23 (table 1). Sensitivity analyses demonstrated that limiting the age to 30 did not alter any results. Two-thirds of participants were females, one-third were males, and less than 2% were defined as non-binary or other gender. Around three-quarters identified themselves as heterosexual. Over two-thirds stated they had no partner, and a notable fifth lived in a monogamous relationship. In the median, participants spent 2.3 hours weekly across two dating apps, predominantly Tinder and Bumble. The median PODAUS score was 9.0 (IQR range: 7–11). 10% of participants reported stalking on dating apps, and 75% deleted and reinstalled ODAs. 71% showed at least mild signs of depression, and 9% showed signs of alcohol use disorder. The most used illegal substance was cannabis, followed by cocaine, MDMA/Ecstasy and (meth-)amphetamines. Regarding ODAs as a gateway for substance use, 7% of participants reported that illegal substances were offered for sale on ODAs, 2% started illegal substance use, and 4% used more illegal substances because of ODAs. 11% of the participants reported at least one STI in their lifetime, and 4% reported at least one STI in the past 12 months, with chlamydia being the most prevalent STI (see online supplemental figure SIV for information on STI diagnoses).

### PODAUS: tests of dimensionality and reliability

The German 6-item PODAUS showed excellent fit in the CFA (χ²(9)=26.63, p≤0.001, Comparative Fit Index (CFI)=0.99, Tucker–Lewis Index (TLI)=0.98, Root Mean Square Error of Approximation (RMSEA)=0.06 and Standardized Root Mean Square Residual (SRMR)=0.02). PODAUS also showed good internal consistency (α=0.73) at baseline and acceptable test–retest reliability (*r_s_*=0.54, p*≤*0.01).

### PODAUS: MI

Results of the MI tests across genders can be found in online supplemental table SV. Since the group size for non-binary and ‘other’ gender categories was very small (n=15), we limited MI testing to participants identifying as male or female. Configural invariance (M2) was established, indicating that the latent structure of the construct is invariant across both genders, thus supporting the idea of an identical factor number and pattern of factor-item relations. Metric invariance (M3) was confirmed, suggesting that all genders responded to items similarly, resulting in comparable ratings. Scalar invariance (M4) provided evidence that participants with the same value on the latent construct of problematic ODA use have equal values on the observed.^57^

### Correlational analysis

Higher PODAUS scores were positively correlated with symptoms of depression (r_s_=0.23, p<0.01) and impulsivity (r_s_=0.16, p<0.01; displayed in online supplemental table SI; also see online supplemental figure SVI for a heatmap of all pairwise correlations). Regarding substance use, no significant associations were found. In terms of sexual behaviour and health, significant correlations were found between PODAUS scores and lifetime STI prevalence (r_s_=0.11, p<0.01). Regarding ODA behaviour, PODAUS scores were significantly and strongly positively correlated with the PTUS score (r_s_=0.65, p<0.01). Additionally, higher intensity of PODAUS was significantly correlated with a higher number of times that participants reported having deleted and reinstalled ODAs (r_s_=0.30, p<0.01) and more stalking experiences on ODAs (r_s_=0.10, p<0.01). Regarding ODA as a potential gateway for substance use, higher PODAUS scores were significantly correlated with more participants reporting (a) that illegal substances were offered for sale on ODAs (r_s_=0.10, p<0.01) and (b) higher substance use due to ODA use (r_s_=0.12, p<0.01).

### Multiple regression analysis

See table 2 for significant multiple regression outcomes with covariates age and genders (online supplemental table SVII also includes non-significant results.

**Table 2:** Summary of multiple regression results highlighting only significant effects of PODAUS on outcome variables (full table can be found in online supplemental table SVII)

Outcome	Type	Predictors	B	CI (LL, UL)	R²	f²/ratios	P-value
Mental health							
Depression (n=911)	OLS	PODAUS	0.43	0.31, 0.54	0.07	0.07	**<0.001**
		Age	−0.12	−0.18, 0.01			0.093
		Gender_f_	0.85	0.17, 1.54			0.015
		Gender_nbo_	0.56	−2.01, 3.14			0.667
Impulsivity (n=903)	OLS	PODAUS	0.32	0.18, 0.46	0.03	0.03	**<0.001**
		Age	−0.12	−0.24, 0.00			0.051
		Gender_f_	0.03	−0.82, 0.88			0.947
		Gender_nbo_	2.35	−0.95, 5.65			0.162
Sexual behaviour							
Sexual partner count (n=864)	OLS	PODAUS	0.01	0.00, 0.01	0.03	0.03	**0.002**
		Age	0.01	0.02, 0.01			**<0.001**
		Gender_f_	−0.02	−0.05, 0.01			0.286
		Gender_nbo_	−0.01	−0.13, 0.11			0.854
Sexual health							
Lifetime STI prevalence (n=923)	PLR	PODAUS	1.12	1.05, 1.19	0.10	1.12*	**0.001**
		Age	1.12	1.07, 1.17		1.12	0.017
		Gender_f_	0.99	0.67, 1.48		0.99	0.908
		Gender_nbo_	2.10	0.51, 5.83		2.10	0.246
12-month STI prevalence (n=923)	PLR	PODAUS	1.17	1.06, 1.28	0.09	1.17†	**0.001**
		Age	1.10	1.01, 1.18		1.10	0.017
		Gender_f_	0.60	0.31, 1.16		0.60	0.125
		Gender_nbo_	2.96	0.47, 10.44		2.96	0.148

Bold highlights significant outcomes (p<.01)

* Incidence rate ratio.

† Odds ratio.

LL, lower limits; nbo, non-binary and other; OLS, ordinary least squares; PLR, Polynomial Linear Regression; PODAUS, Problematic Online Dating Apps Use Scale; STI, sexually transmitted infection; UL, upper limits.

#### Mental health outcomes

Higher PODAUS scores significantly predicted poorer mental health outcomes with small effect sizes. Specifically, PODAUS predicted higher depression scores (B=0.43, p<0.001), with age (B=−0.12, p<0.01) as a significant covariate. Higher PODAUS scores also predicted higher impulsivity in the BIS-15 (B=0.32, p<0.001*).*

#### Substance use outcomes

All substance use outcomes did not yield significant results (p<0.01) in the multiple linear regression results.

#### Sexual behaviour/sexual health outcomes

Higher PODAUS scores significantly predicted a higher number of sexual partners in the past 3 months (B=0.07, p<0.01), with age as a significant covariate (B=0.05, p<0.001). Regarding sexual health, an increase in PODAUS scores predicted a 1.12-fold increase in the Lifetime STI Prevalence (p<0.001) as well as a 1.17-fold increase in the 12-month prevalence of STIs (p<0.01*).*

## Discussion

This study demonstrates significant associations between higher intensity of problematic ODA use and adverse mental and sexual health outcomes and a higher number of sexual partners in 923 university students.

### Psychometric properties of the German PODAUS

The PODAUS demonstrated excellent internal consistency and acceptable test-retest reliability over two weeks. Moreover, the strong association between PODAUS and PTUS underlines its conceptual validity. MI was demonstrated at all levels (configural, metric and scalar) across genders, confirming that the PODAUS measures problematic ODA use equivalently and is understood similarly by both male and female students.

### General findings

In our sample, Tinder was the most used ODA, consistent with global statistics reporting it as the most favoured global ODA, with 4.4 million downloads as of February 2024.^58^ Notably, 17% of our sample identified as bisexual and 5% as homosexual, percentages exceeding the typical 1%–3% reported in Switzerland and other Western nations.^59^,^61^ Nevertheless, our numbers are consistent with a 2024 survey comprising 26 countries reporting that 17% of respondents identified as non-heterosexual, with younger individuals, particularly those belonging to Generation Z (born between 1996 and 2012), being the most likely to identify as non-heteronormative sexual orientations, reflecting the primary age range of our sample.^62^ However, these results should be interpreted with caution, as our study only captured self-reported sexual orientation, not past lived sexual or romantic experiences, and 64% of the sample identified as female.

Participants’ primary motivations for ODA use were sexual (‘finding a sexual partner’) and romantic (‘finding a partner for life’), consistent with prior research on Dutch emerging adults’ motivation for Tinder use.^63^ These motivations reflect the concept of social gratification, as emerging adults often use online platforms to meet developmental needs related to establishing romantic and sexual relationships and thus fulfilling a social versus physical need.^64^ Interestingly, the third most commonly reported motivation for ODA use was ‘confirmation for appearance’, a form of self-worth validation. This motivation reflects the fulfilment of psychosocial needs related to enhancing self-esteem and feeling better about oneself, a key driver identified in prior research.^63^ However, rather than assuming ODAs effectively satisfy these needs, it is crucial to consider whether they may leave users feeling unfulfilled, perpetuating a cycle of dissatisfaction and excessive use. This perspective underscores the potential for problematic usage patterns and associated adverse health outcomes, as suggested by prior research.^25^ Future studies are needed to determine whether the pursuit of these gratifications through ODAs ultimately contributes to adverse mental health effects.

### Mental health outcomes

As expected, our study revealed a significant association between higher PODAUS scores and adverse mental health outcomes, such as increased levels of depression and impulsive behaviour. One interpretation of these findings is that problematic ODA use may contribute to poorer mental health, possibly exacerbating feelings of loneliness and social isolation due to the superficial and transient nature of interactions on these platforms.^9 10^ Alternatively, poorer mental health may contribute to more intensive ODA use, potentially serving as a coping mechanism. This highlights the necessity for longitudinal studies to elucidate the directionality of these effects. Notably, age and gender were significant covariates in these analyses, with younger age and female gender associated with higher depression scores. This is in line with recent research highlighting an increase in psychological symptoms such as depression and anxiety, particularly among young women.^65 66^

### Sexual health outcomes

The study demonstrated significant associations between higher PODAUS scores and risky sexual behaviours and sexual health, such as a higher number of sexual partners and increased STI prevalence, respectively. This might be underlined by the fact that a higher PODAUS was not only associated with a higher lifetime but also a higher 12-month STI prevalence, highlighting the potential of ODAs to facilitate unsafe sexual practices.^13^,^15^ While the number of sexual partners is commonly used as a proxy for sexual risk, it is a limited measure, as it does not account for factors like condom use, sexual health communication or the context of encounters, which significantly influence STI risk.^67^ Future research should keep this in mind to provide a more comprehensive understanding of sexual risk and its link to problematic ODA use.

It is important to note that age was a significant covariate in predicting the number of sexual partners and STI prevalence, with older students more likely to report higher numbers of sexual partners and higher STI prevalence. This finding necessitates attention from sexual health educators and practitioners to address the risks associated with ODA use, especially among older students. However, older participants might have had more time to acquire STIs and possibly also used ODAs for longer.

### Substance use outcomes

We did not find any statistically significant associations between problematic ODA use and any substance use outcomes, contrary to our hypothesis. There are multiple possible explanations for this lack of association. First, it may reflect a true absence of a relationship, which aligns with some of the existing literature.^68^ Second, behavioural addictions and substance use disorders may have inherently different etiopathogeneses.^69^ Third, self-reported substance use is known to be underreported by 30%–60% of young adults participating in surveys.^43^ Prior research indicates that ODA use is associated with substance use primarily in the context of sexualised settings, suggesting that future studies should further investigate this aspect.^70^ Finally, our sample consisted predominantly of young females, a group shown to have lower substance use rates and different ODA use motivations than their male counterparts.^42 43 71^ Nevertheless, our findings suggest that ODA use may act as a gateway to substance use for some individuals, as indicated by 7% of participants who reported being offered illegal substances on ODAs, 4% who reported an increase in illegal substance use due to ODA use, and 2% who started using illegal substances because of ODAs. The connection between substance use and dating app use is complex and multifaceted; ODAs can create environments conducive to substance use, influenced by broader sociocultural factors, such as peer pressure, developmental stages or psychosocial stressors, which can shape the individual’s relationship with both apps and substances.

This situation, coupled with the positive correlations between higher intensity of problematic ODA use and stalking experiences, underlines crucial public health interventions and prevention strategies for those most at risk. ODA users, especially younger students, must be informed about safer use practices regarding their mental health, substance use behaviour and sexual health when navigating the digital world.

### Strengths and limitations

This study leveraged a large cohort of Swiss university students and employed a variety of validated psychometric instruments to comprehensively assess mental health, sexual health and behavioural outcomes. In addition to examining problematic ODA use, the study provided detailed insights into usage patterns, motivations and time spent on these platforms, offering a nuanced understanding of ODA-related behaviours. Nevertheless, this study has several limitations, including its cross-sectional design and reliance on self-reported data, which has been shown to present biases and underreporting, specifically regarding substance use.^43^ Given that ODA use occurs within a complex sociocultural context, it is crucial not to oversimplify the relationship between ODA use and related behaviour. As this study is cross-sectional, causal inferences or temporal relationships cannot be established. Future research should employ longitudinal designs including open questions and qualitative research, objective substance use assessments, such as toxicological hair analysis, to decipher the interconnection of problematic ODA use and mental and sexual health outcomes, further explore the mechanisms underlying these associations, and assess possible long-term effects of problematic ODA use.

Shorter time frames should be considered when investigating ODA use patterns, related behaviours and health outcomes. In this study, we defined active ODA users as those who reported ODA use in the past 12 months. Future research might adopt shorter and more consistent timeframes across all outcomes. To improve the generalisability of findings, future research should prioritise including more diverse populations, as current studies, including ours, predominantly sample females from higher-income countries.^34^ Finally, research should focus on individuals with severe problematic ODA use to better understand the underlying mechanisms of ODA-related behaviours and their associated outcomes.^72^

### Implications

In today’s highly digital world, where young people spend substantial time online, understanding and addressing behavioural addictions is critical. Our study highlights that problematic ODA use is significantly associated with adverse mental and sexual health outcomes in university students, including elevated depressive symptoms, impulsivity, a higher number of sexual partners and increased lifetime and 12-month STI prevalence rates. Despite these risks, a substantial gap in care persists, leaving affected individuals with limited support options. These findings underscore the urgent need for tailored, holistic interventions that integrate problematic ODA use into mental health, substance use and sexual healthcare frameworks. Strengthening social support networks, advancing digital literacy and promoting healthier ODA use practices are essential to mitigate possible negative impacts of this behaviour and enhance student well-being. By providing platforms for safe interaction, encouraging protective behaviour and discouraging problematic and prolonged use, and offering educational initiatives that enhance self-efficacy in navigating dating environments, ODAs could serve as critical tools for navigating the often-complex landscape of young adult relationships.

Finally, the German version of the PODAUS proved to be a reliable and valid tool for identifying problematic ODA use in a high-user group of emerging adults, with robust MI across genders. Its practical application in clinical settings enables brief and efficient screening, supporting early identification of at-risk individuals and timely interventions to reduce adverse outcomes.

## Supplementary material

10.1136/bmjph-2025-002569 online supplemental file 1

## Data Availability

Data are available on reasonable request. No data are available.
