# Supplementary material for: Relations of problematic online dating app use with mental and sexual health: a cross-sectional study in Swiss university students
Source: BMJ Public Health. 2025 Sep 8;3(2):e002569. doi: 10.1136/bmjph-2025-002569 (PMC12421148; doi:10.1136/bmjph-2025-002569)
Supplement: online supplemental file 1 [file bmjph-3-2-s001.docx]

**Supplementary Materials**

| **Supplementary Table SI.** Reliability, Spearman’s pairwise correlations with PODAUS score at Baseline | | |
| --- | --- | --- |
|  | **α** | **r_s_** |
| **Demographics (*N*=923)** |  |  |
| Age |  | -.04 |
| Sex |  | -.02 |
| Gender |  | -.02 |
| Sexual orientation |  | .01 |
| **Problematic ODA use (*N*=923)** |  |  |
| Time spent on ODAs (in h) |  | .33** |
| Baseline: Problematic Dating App Use (PODAUS; *N*=923) | .73 | 1.00 |
| Follow-up: Problematic Dating App Use (PODAUS; *_N_*=275) | .77 | .54** |
| Problematic Tinder Use (PTUS; *n*=630) | .76 | .65** |
| **Additional ODA questions (*N*=923)** |  |  |
| Experienced stalking on ODAs |  | .10** |
| Deleted and reinstalled ODAs |  | .30** |
| Illegal substances were offered for sale on ODAs |  | .10** |
| Started illegal substance use because of ODAs |  | .07* |
| Increased illegal substance use because of ODAs |  | .12** |
| **Mental Health** |  |  |
| Depression (PHQ-9, *n*=911) | .85 | .23** |
| Impulsivity (BIS-15, *n*=903) | .76 | .16** |
| **Substance use** |  |  |
| Intensity of alcohol use (AUDIT, *n*=641) | .71 | .09* |
| Nicotine (Cigarettes/day, *n=*890) |  | .08* |
| **12-month prevalence (*n*=890)** |  |  |
| Cannabis |  | .03 |
| Cocaine |  | .03 |
| Ecstasy/ MDMA |  | .03 |
| Amphetamine/ Methamphetamine |  | -.02 |
| Nicotine |  | .09** |
| **30-day frequency (*n*=890)** |  |  |
| Cannabis |  | .04 |
| Cocaine |  | .07* |
| Ecstasy/ MDMA |  | .01 |
| Amphetamine/ Methamphetamine |  | -.04 |
| **Sexual Health** |  |  |
| Lifetime STI Prevalence (*N*=923) |  | .11** |
| 12-Month STI Prevalence (*N*=923) |  | .09** |
| Sexual Partner count (in past 3 months; *n*=864) |  | .11** |
| Sexlife Happiness (*n*=923) |  | .00 |
| Sexual Self-esteem (SSES; *n*=885) | .37 | .02 |
| ***Note.*** **p* < 0.05, ***p* < 0.01, α=Cronbach’s alpha, r*_s_*=Spearman’s rank correlation coefficient for pairwise comparisons with Problematic Online Dating App Use Scale (PODAUS) score, ODA=online dating app, STI=sexually transmitted infection | | |

| **Supplementary Table SII.** German version of the Problematic Online Dating App Use Scale (PODAUS) |
| --- |
| **Items** |
| 1. Meine Gedanken kreisen ständig um Online Dating Apps. |
| 2. Online Dating Apps sind für mich eine Möglichkeit, meine Probleme zu vergessen. |
| 3. Ich verbringe mehr Zeit auf Online Dating Apps als ich eigentlich vorhatte. |
| 4. Wenn ich längere Zeit keine Online Dating Apps verwenden kann (aus technischen, beruflichen oder sozialen Gründen), werde ich unruhig und nervös. |
| 5. Ich habe andere Hobbies oder Freizeitaktivitäten aufgegeben oder stark eingeschränkt, um mehr Zeit auf Online Dating Apps verbringen zu können. |
| 6. Ich habe bereits versucht, Online Dating Apps weniger zu benutzen, habe es aber nicht geschafft. |

| **Supplementary Table SIII.** Descriptive statistics and English translation of PODAUS items (N=923). | | | | | | |
| --- | --- | --- | --- | --- | --- | --- |
| **Component** | **Item** | **M** | ***SD*** | **Skew** | **Kurtosis** | **Item-total cor.** |
| Salience | My thoughts are constantly revolving around dating apps. | 1.35 | .58 | 1.52 | 6.28 | .49 |
| Mood Modification | Dating apps are a way for me to forget my problems. | 1.87 | .88 | .44 | -1.13 | .40 |
| Tolerance | I spend more time on dating apps than I intended to. | 2.21 | .96 | .05 | -1.20 | .47 |
| Withdrawal | If I can’t use dating apps for a long time (for technical, professional, or social reasons), I become restless and nervous. | 1.25 | .55 | 2.28 | 4.85 | .58 |
| Conflict | I have given up or greatly reduced other hobbies or leisure activities to spend more time on dating apps. | 1.22 | .51 | 2.48 | 6.28 | .49 |
| Relapse | I have tried to cut down my dating apps use but have not succeeded. | 1.46 | .70 | 1.34 | .98 | .53 |
| Total score | | 9.36 | 2.81 | .90 | .35 |  |
| *Note.* PODAUS=Problematic Online Dating App Use Scale). English translation of the German original version. Items are answered on a 4-Point scale (1=do not agree to 4=agree completely) | | | | | | |

**Supplementary Fig. SIV.** Absolute numbers of Sexually transmitted Infections (STI) prevalence rates (*N*=923).


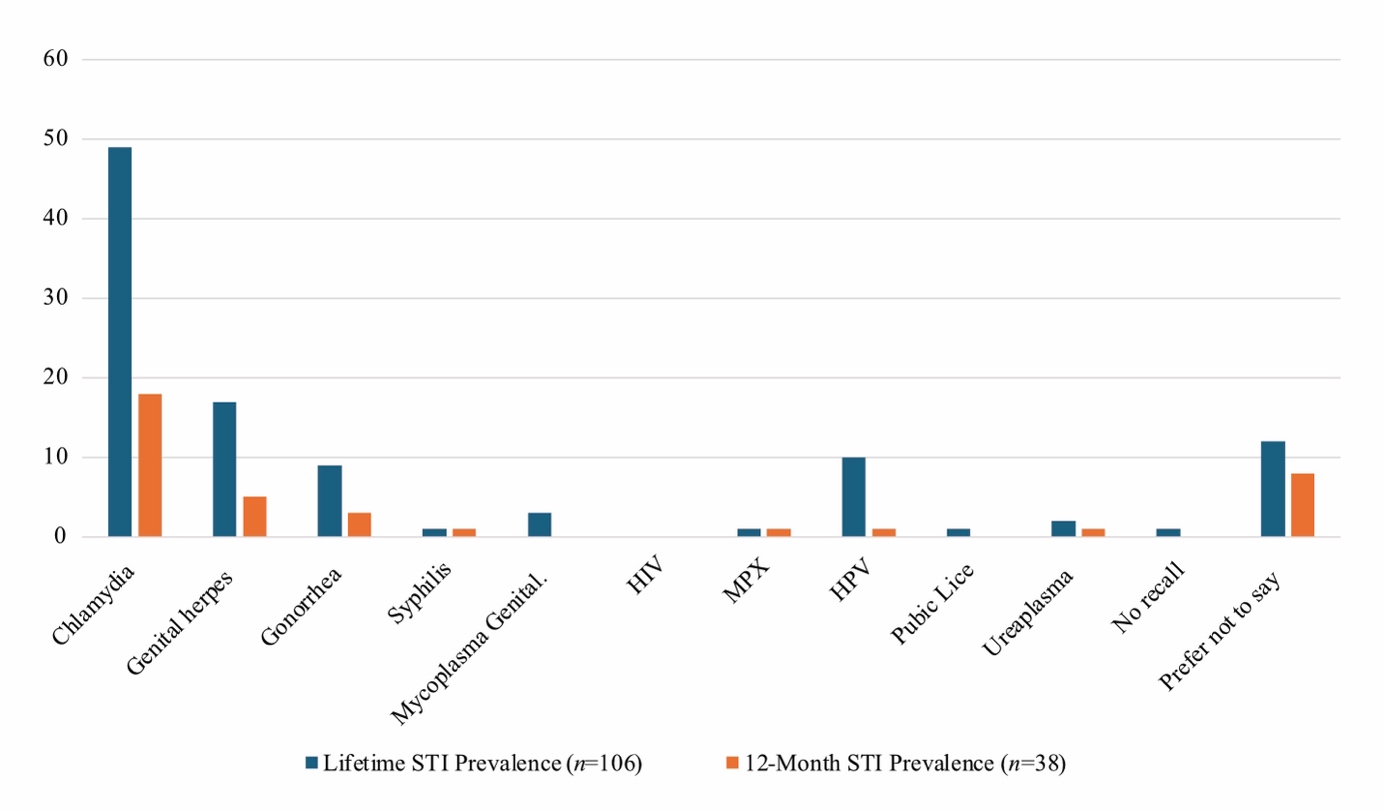


| **_Supplementary Table SV.** Evaluations of multigroup measurement invariance (MGCFA MI) across genders for the PODAUS (*N*=908). | | | | | | | | | | | | | |
| --- | --- | --- | --- | --- | --- | --- | --- | --- | --- | --- | --- | --- | --- |
| Model | Type of test | Compared with | χ^2^  p | df | RMSEA | CFI | TLI | SRMR | Δdf | ΔCFI | ΔRMSEA | ΔSRMR | Decision |
| M1a | Males (*n*=316) |  | 10.7 .299 | 9 | 0.024 [0.000, 0.070] | 0.994 | 0.991 | 0.026 |  |  |  |  |  |
| M1b | Females (*n*=592) |  | 18.3 .032 | 9 | 0.042 [0.012, 0.069] | 0.981 | 0.969 | 0.025 |  |  |  |  |  |
| M2 | Configural invariance |  | 28.9 .049 | 18 | 0.036 [0.001, 0.060] | 0.970 | 0.966 | 0.026 |  |  |  |  |  |
| M3 | Metric invariance | M2 | 38.8 .021 | 23 | 0.039 [0.015, 0.060] | 0.979 | 0.973 | 0.044 | 5 | -.007 | .018 | .018 | Accept |
| M4 | Scalar invariance | M3 | 76.9 <.001 | 28 | 0.062 [0.046, 0.079] | 0.935 | 0.930 | 0.053 | 5 | -.044 | .023 | .009 | Accept |
| *Note:* χ^2^*_=_*Chi-square statistic including p-values, df*=*degrees of freedom*,* RMSEA*=*Root Mean Square Error of Approximation*,* CFI*=*Comparative Fit Index*,* TLI*=*Tucker Lewis Index, SRMR*=*Standardized Root Mean Square Error of Approximation*,* Δ*=*Change in statistical values. | | | | | | | | | | | | | |

**Supplementary. Fig. SVI.** Heatmap of Spearman’s pairwise complete correlations


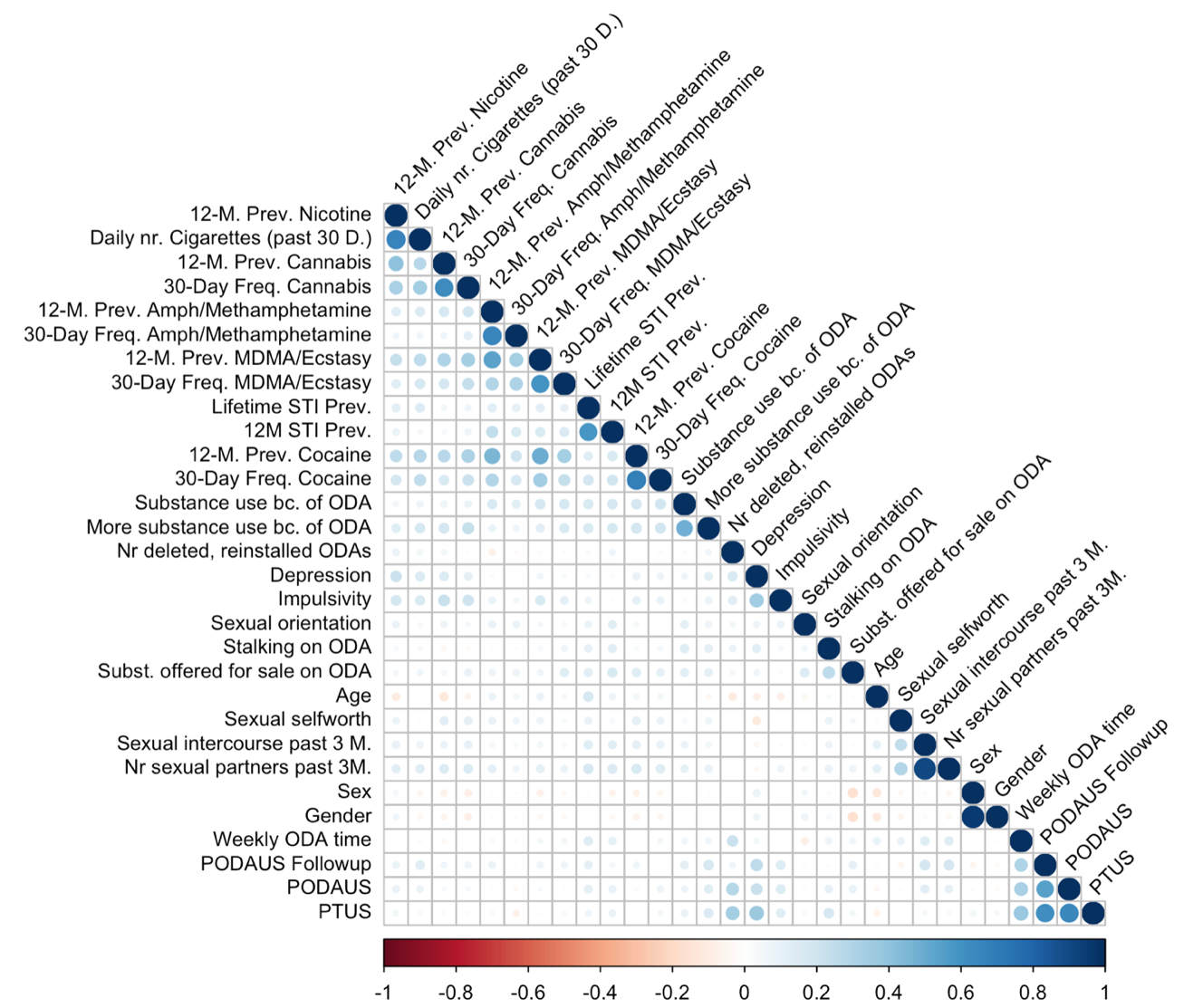


| **Supplementary Table SVII.**  Summary of multiple regression results of all outcomes with PODAUS as predictor. | | | | | | | |  |
| --- | --- | --- | --- | --- | --- | --- | --- | --- |
| **Outcome** | **Type** | **Predictors** | **B** | **CI (LL, UL)** | ***R²*** | ***f²*** | ***p*** | |
| **Mental health** | | | | | | | | |
| Depression (*n*=911) | OLS | PODAUS  Age  Gender_f_  Gender_nbo_ | 0.43  -0.12  0.85  0.56 | 0.31, 0.54  -0.18, 0.01  0.17, 1.54  -2.01, 3.14 | .07 | .07 | **<.001**  .093  .015  .667 | |
| Impulsivity (*n*=903) | OLS | PODAUS  Age  Gender_f_  Gender_nbo_ | 0.32  -0.12  0.03  2.35 | 0.18, 0.46  -0.24, 0.00  -0.82, 0.88  -0.95, 5.65 | .03 | .03 | **<.001**  .051  .947  .162 | |
| **Sexual Behavior** | |  |  |  |  |  |  | |
| Sexual partner count (*n*=864) | OLS | PODAUS  Age  Gender_f_  Gender_nbo_ | 0.01  0.01  -0.02  -0.01 | 0.00, 0.01  0.02, 0.01  -0.05, 0.01  -0.13, 0.11 | .03 | .03 | **.002**  **<.001**  .286  .854 | |
| Sexual Self-esteem (*n*=885) | OLS | PODAUS  Age  Gender_f_  Gender_nbo_ | 0.04  0.01  -0.31  -1.37 | -0.04, 0.13  -0.05, 0.07  -0.82, 0.20  -3.41, 0.67 | .00 | .00 | .298  .913  .310  .194 | |
| Sexlife satisfaction (*n*=885) | OLS | PODAUS  Age  Gender_f_  Gender_nbo_ | -0.24  -0.31  7.20  14.59 | -0.90, 0.44  -0.60, 0.50  3.14, 11.18  -1.21, 30.64 | .02 | .02 | .498  .856  **<.001**  .070 | |
| **Sexual Health** | | | | | | | | |
| Lifetime STI prevalence (*n*=923) | PLR | PODAUS  Age  Gender_f_  Gender_nbo_ | 1.12  1.12  0.99  2.10 | 1.05, 1.19  1.07, 1.17  0.67, 1.48  0.51, 5.83 | .10 | 1.12^a^  1.12  0.99  2.10 | **.001**  .017  .908  .246 | |
| 12-Month STI prevalence (*n*=923) | PLR | PODAUS  Age  Gender_f_  Gender_nbo_ | 1.17  1.10  0.60  2.96 | 1.06, 1.28  1.01, 1.18  0.31, 1.16  0.47, 10.44 | .09 | 1.17^a^  1.10  0.60  2.96 | **.001**  .017  .125  .148 | |
| **Substance Use** | | | | | | | | |
| AUDIT (*n*=637) | OLS | PODAUS  Age  Gender_f_  Gender_nbo_ | 0.06  -0.03  -0.97  -0.30 | -0.02, 0.14  -0.09, 0.03  -1.44, -0.5  -2.33, 1.73 | .03 | .03 | .140  .368  **<.001**  .774 | |
| 12-Month prevalence Nicotine (*n*=890) | LogR | PODAUS  Age  Gender_f_  Gender_nbo_ | 0.06  -.05  0.23  0.79 | 1.01, 1.11  0.92, 1.00  0.94, 1.69  0.71, 7.08 | .02 | 1.06^b^  0.96  1.25  2.21 | .015  .031  .131  .167 | |
| Daily Nicotine intake  (Nr of cigarettes/day; *n*=890) | OLS | PODAUS  Age  Gender_f_  Gender_nbo_ | 0.05  0.01  -0.39  -0.61 | -0.02, 0.11  -0.04, 0.05  -0.80, 0.01  -2.23, 1.00 | .01 | .01 | .182  .832  .056  .456 | |
| 12-Month Prevalence  Cannabis (*n*=887) | LogR | PODAUS  Age  Gender_f_  Gender_nbo_ | 0.00  -0.08  -0.40  0.73 | 0.95, 1.05  0.88, 0.96  0.50, 0.90  0.67, 7.06 | .03 | 4.79 ^b^  1.00  0.92  0.67 | .935  **<.001**  **.007**  .210 | |
| 12-Month prevalence Cocaine (*n*=888) | LogR | PODAUS  Age  Gender_f_  Gender_nbo_ | 0.04  0.03  -0.47  -0.22 | 0.95, 1.13  0.97, 1.09  0.38, 1.04  0.04, 4.34 | .01 | 1.04^b^  1.03  0.62  0.80 | .355  .255  .066  .838 | |
| 12-Month prevalence MDMA (*n*=889) | LogR | PODAUS  Age  Gender_f_  Gender_nbo_ | 0.04  0.05  -0.52  1.82 | 0.96, 1.13  1.00, 1.11  0.36, 0.99  1.75, 19.96 | .03 | 1.04^b^  1.05  0.59  6.17 | .320  .056  .044  **.003** | |
| 12-Month prevalence (Meth-) amphetamine (*n*=889) | LogR | PODAUS  Age  Gender_f_  Gender_nbo_ | -0.05  0.09  -0.07  1.07 | 0.82, 1.09  1.02, 1.17  0.43, 2.11  0.15, 17.57 | .01 | 0.95^b^  1.10  0.93  2.91 | .485  **.006**  .856  .330 | |
| 30-day consumption frequency Cannabis (*n*=890) | OLS | PODAUS  Age  Gender_f_  Gender_nbo_ | -0.02  -0.04  -0.68  -0.28 | -0.09, 0.06  -0.10, 0.01  -1.13, -0.22  -2.09, 1.54 | .01 | .01 | .673  .150  **.004**  .766 | |
| 30-day consumption frequency Cocaine (*n*=890) | OLS | PODAUS  Age  Gender_f_  Gender_nbo_ | 0.00  0.00  0.00  -0.07 | -0.01, 0.02  -0.01, 0.01  -0.06, 0.07  -0.34, 0.02 | .00 | .00 | .401  .585  .925  .618 | |
| 30-day consumption frequency MDMA (*n*=890) | OLS | PODAUS  Age  Gender_f_  Gender_nbo_ | 0.00  0.00  -0.02  -0.05 | -0.00, 0.01  -0.00, 0.01  -0.05, 0.02  -0.20, 0.10 | .00 | .00 | .312  .209  .432  .531 | |
| 30-day consumption frequency (Meth-/ amphetamine (*n*=890) | OLS | PODAUS  Age  Gender_f_  Gender_nbo_ | -0.00  0.01  -0.08  -0.03 | -0.03, 0.02  -0.01, 0.03  -0.23, 0.07  -0.63, 0.57 | .00 | .00 | .785  .307  .287  .927 | |
| *Note.* LL=Lower limits, UL=Upper limits, WLS=Weighted least squares, OLS=Ordinary least squares, LogR=Logarithmic regression, nbo=non-binary and other, ^a=^Incidence rate ratio, ^b=^Odd’s ratio, bold highlights significant outcomes (*p*<.01) | | | | | | | |  |
